# TExCNN: Leveraging Pre-Trained Models to Predict Gene Expression from Genomic Sequences

**DOI:** 10.3390/genes15121593

**Published:** 2024-12-12

**Authors:** Guohao Dong, Yuqian Wu, Lan Huang, Fei Li, Fengfeng Zhou

**Affiliations:** 1 Key Laboratory of Symbolic Computation and Knowledge Engineering of Ministry of Education, Jilin University, Changchun 130012, China; donggh2000@126.com (G.D.); wyq_sylvia@163.com (Y.W.); huanglan@jlu.edu.cn (L.H.); comeherewinter@gmail.com (F.L.); 2 College of Computer Science and Technology, Jilin University, Changchun 130012, China; 3 College of Software, Jilin University, Changchun 130012, China

**Keywords:** gene expression prediction, DNA sequence, pre-trained model, regression task, deep learning

## Abstract

Background/Objectives: Understanding the relationship between DNA sequences and gene expression levels is of significant biological importance. Recent advancements have demonstrated the ability of deep learning to predict gene expression levels directly from genomic data. However, traditional methods are limited by basic word encoding techniques, which fail to capture the inherent features and patterns of DNA sequences. Methods: We introduce TExCNN, a novel framework that integrates the pre-trained models DNABERT and DNABERT-2 to generate word embeddings for DNA sequences. We partitioned the DNA sequences into manageable segments and computed their respective embeddings using the pre-trained models. These embeddings were then utilized as inputs to our deep learning framework, which was based on convolutional neural network. Results: TExCNN outperformed current state-of-the-art models, achieving an average R^2^ score of 0.622, compared to the 0.596 score achieved by the DeepLncLoc model, which is based on the Word2Vec model and a text convolutional neural network. Furthermore, when the sequence length was extended from 10,500 bp to 50,000 bp, TExCNN achieved an even higher average R^2^ score of 0.639. The prediction accuracy improved further when additional biological features were incorporated. Conclusions: Our experimental results demonstrate that the use of pre-trained models for word embedding generation significantly improves the accuracy of predicting gene expression. The proposed TExCNN pipeline performes optimally with longer DNA sequences and is adaptable for both cell-type-independent and cell-type-dependent predictions.

## 1. Introduction

Gene expression can be understood as the process of producing observable phenotypes from coding genes. Genes express phenotypes under specific environmental conditions [1], and mRNAs and proteins serve as intermediates in this process [2]. Although mRNA abundance is not strictly equivalent to gene expression levels, this study uses mRNA abundance as a proxy for gene expression [3,4].

DNA sequences encode the temporal and spatial patterns of gene expression levels, which are recognized and interpreted by transcription factors (TFs) [5]. Enhancers and promoters within DNA sequences regulate gene expression by interacting with these TFs [6]. Previous studies have applied machine learning methods to investigate the interactions between gene regulatory elements [7].

Developing a quantitative model to accurately predict gene expression levels from genomic sequences provides valuable insights into how genomic variations influence gene expression [8]. Recent studies [8,9,10,11,12,13,14,15] have leveraged deep learning approaches to predict gene expression levels directly from DNA sequences.

Xpresso [9] is an early cell-type-independent gene expression predictor that models median gene expression levels as a regression task. Its architecture consists of two convolutional neural network (CNN) layers, a max-pooling layer, and two fully connected layers. Its results demonstrate that gene expression can be predicted from DNA sequences near transcription start sites (TSS). DeepLncLoc [15] is a text CNN-based predictor that utilizes a 3-mer and Word2Vec-based [16] word vector generator to transform sequences into word embeddings, enhancing the model’s ability to capture critical features. DNAPerceiver [10] combines CNN and Transformer architectures [17] to overcome the limitations of CNNs in handling long-range interactions. However, Xpresso, DeepLncLoc, and DNAPerceiver are limited to processing sequences of only 10,500 bp, which constrains their accuracy for more comprehensive predictions.

Enformer [11] is one of the largest models, including 7 CNN layers and 11 Transformer layers, capable of processing input sequences up to 196 kb in length. Enformer outputs predictions for over 5000 genomic features, including transcription factor binding, histone modifications, and chromatin accessibility. While Enformer is highly effective in handling long input sequences, its training process demands substantial computational resources. Furthermore, since Enformer does not directly perform gene-based, cell-type-independent prediction, it cannot be directly compared to the methods presented in this paper, nor to methods like Xpresso.

Additionally, existing models face challenges in fully capturing the effects of distant enhancers on gene regulation [18]. As the sequence length extends beyond tens of thousands of base pairs, the newly included regions contribute minimally to prediction accuracy, underscoring a significant limitation of current approaches.

The encoding of DNA sequences is critical for achieving high prediction accuracy. Most models use one-hot encoding, where each nucleotide (A/C/G/T) is represented as a sparse vector. This method fails to capture relationships between neighboring nucleotides. DeepLncLoc [16] partially addresses these limitations by using a 3-mer and Word2Vec-based embedding, but it is trained on limited datasets rather than the entire human genome. There remains significant room for improvement in word embedding techniques for DNA sequence pre-prossing.

This study introduces a new framework, TExCNN, which leverages pre-trained models DNABERT [19] and DNABERT-2 [20] to generate word embeddings for DNA sequences. By utilizing these pre-trained models, TExCNN can effectively capture inherent features and patterns within DNA sequences.

We benchmarked TExCNN against the text CNN-based DeepLncLoc model, demonstrating its superior performance. Additionally, we extended the dataset in two ways: first, by increasing the input sequence length from 10,500 bp to 50,000 bp, and second, by expanding the labels from a single value to 57 distinct values. We trained TExCNN on this expanded dataset to further demonstrate its capabilities.

The remainder of this paper is structured as follows: we first describe the datasets and pre-trained models used in this study. Next, we present the TExCNN framework, including the word vector generation process and model architecture. We then discuss experimental results, followed by a conclusion that summarizes the key findings.

## 2. Datasets and Methods

### 2.1. Xpresso Dataset

The Xpresso dataset was derived from RNA-seq gene expression data provided by the Epigenomics Roadmap Consortium [21]. The raw data included gene expression levels across 57 tissues and cell types. The Xpresso authors [9] used the median values of these expression levels as labels for model training and made Xpresso a cell-type-independent model.

To create the input sequences, the Xpresso authors centered the CAGE peak of each gene, and extracted 10,000 bp upstream and 10,000 bp downstream sequences from the human genome (hg38) [22]. After hyperparameter optimization, the final input sequences consisted of 7000 bp upstream and 3500 bp downstream of the CAGE peak.

MRNA half-life is a key factor in gene regulation and disease progression [23]. Therefore, the Xpresso authors further incorporated half-life data for each gene, which consisted of eight values explaining the variation in mRNA half-life.

### 2.2. DeepLncLoc Dataset

The DeepLncLoc dataset [15] was expanded upon the Xpresso dataset by incorporating transcription factor binding data [24]. For each gene, the dataset included 181 binary values, where a value of “1” indicated that the gene was a target of a transcription factor, and a value of “0” indicated it was not.

### 2.3. Extended Dataset

The Xpresso paper [9], reported that the model performs best when the input sequence included 7000 bp upstream and 3500 bp downstream of the promoter. Howerver, enhancers can regulate gene expression from distances spanning tens of thousands of base pairs [25]. To further explore the effect of distal enhancers on gene expression, the model should incorporate more distant sequences as inputs.

Given the constraints of computational resources, we limited the input sequences to a maximum length of 50,000 bp rather than using longer sequences. In the following experiments, we will investigate the impact of sequences from different regions on prediction accuracy.

We extended the Xpresso and DeepLncLoc datasets by increasing the sequence length to include 25,000 bp upstream and 25,000 bp downstream of each gene from the hg38 reference genome. To achieve this, we utilized the CAGE peak file provided by the authors of Xpresso along with Bedtools. Additionally, we retained the 57 gene expression values across different tissues and cells (including one universal human reference expression value) from the raw dataset of Xpresso for more detailed analysis.

**Figure 1:**
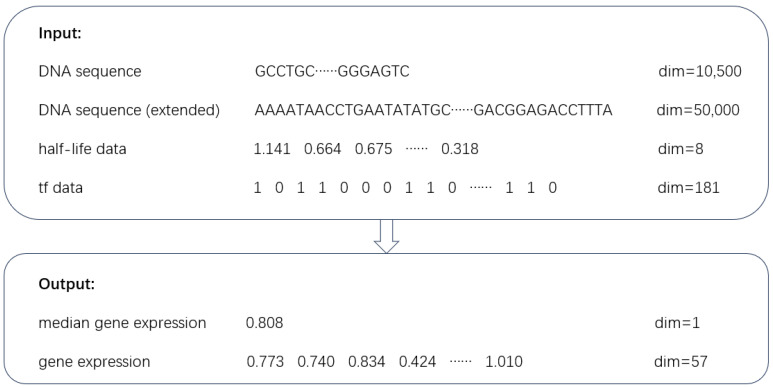
The datasets used in this study. The features include DNA sequences, half-life data, and transcription factor target data. The labels correspond to gene expression values.

Figure 1 illustrates the features and labels of the extended dataset, which include DNA sequences, mRNA half-life data, transcription factor target data, and gene expression values.

### 2.4. Pre-Trained Models

DNA sequences share structural similarities with human language, making natural language processing (NLP) techniques applicable to genomic data. In recent years, large-scale, deep learning-based pre-trained models like Bidirectional Encoder Representations from Transformers (BERT) [26] have revolutionized the field of NLP. Inspired by these advancements, researchers have developed similar models for the human genome, including DNABERT [19], DNABERT-2 [20], and the Nucleotide Transformer [27].

DNABERT, a BERT-based pre-trained model, has been specifically designed for human genomic data [19]. It consists of 12 Transformer layers, each with 768 hidden units and 12 attention heads. DNABERT uses 6-mer encodings for DNA sequences, generating overlapping 6-mers from a sequence. For example, the sequence “ACGTTCGA” is encoded as [“ACGTTC”, “CGTTCG”, “GTTCGA”]. The maximum input length is 512 tokens, which corresponds to 515 bp. Since DNABERT is a representative pre-trained model trained on the human genome, we have selected it for our pipeline.

DNABERT-2, an extension of DNABERT, improves upon the original by replacing the k-mer encoding with Byte Pair Encoding (BPE) [28]. BPE encodes DNA sequences into words of varying lengths, with an average word length of around 4 bp. This modification increases the maximum input length to approximately 2000 bp while maintaining a 512-word token limit. Additionally, DNABERT-2 has been trained on a multi-species genome. In contrast to the Nucleotide Transformer, which has also been trained on a multi-species genome, DNABERT-2 is much smaller in size but still performes well across many tasks. Therefore, we have selected DNABERT-2 rather than the Nucleotide Transformer in our pipeline.

Although DNABERT and DNABERT-2 have achieved strong performance on the Genome Understanding Evaluation (GUE) classification benchmark [20], these tasks are limited to sequence lengths shorter than 1000 bp. However, predicting gene expression requires input sequences of at least 10,500 bp, exceeding the 512-word limit. Therefore, the fine-tuning techniques used for classification tasks cannot be directly applied to gene expression prediction.

Addtionally, while fine-tuning is an effective method for solving many problems, it requires significant computational resources for each specific downstream task. Moreover, the model parameters that are fine-tuned for one task cannot be directly applicable to other tasks. Therefore, for certain deep learning applications, an alternative approach to fine-tuning should be considered.

### 2.5. Sequence Representations

To overcome the limitations of traditional encoding methods, TExCNN uses the embedding representations from DNABERT and DNABERT-2 as inputs for the subsequent models.

DNABERT processes sequences by tokenizing them into 6-mers, with a maximum input length of 512 tokens (515 bp). In the experiment below, we used sequences of 10,500 bp. For simplicity, we spli them into 20 fragments of 500 tokens (505 bp) and 1 fragment of 495 token (500 bp). The fragments were inputed into DNABERT and transformed into 20 matrices of shape [500, 768] and one matrix of shape [495, 768]. The matrices were then averaged along the first dimension and concatenated to produce a final matrix of size [21, 768]. This process is illustrated in Supplementary Figure S1a.

DNABERT-2 supportes longer input sequences, with lengths up to approximately 2000 bp. To handle even longer sequences, we applied a sliding window approach, splitting the sequence into fragments of 2000 bp with a 500-bp step size. For example, a 10,500-bp sequence was divided into 18 fragments. Each fragment was processed and averaged into a vector of length 768, resulting in a matrix of size [18, 768] after concatenation. This process is illustrated in Supplementary Figure S1b.

The contrast experiment between different pre-processing methods is presented in Appendix A.

### 2.6. TExCNN Model with a 10,500-bp Input Length

Different pre-trained models capture distinct hidden patterns in DNA sequences. Combining sequence representations from multiple models can enhance the final predictive performance.

Our approach uses word vectors generated by both DNABERT and DNABERT-2 as the inputs (Supplementary Figure S2a). These vectors are processed individually through a CNN layer, followed by a batch normalization layer and a dropout layer, before being concatenated. The GELU activation function [29] is applied due to its ability to retain more useful information compared to ReLU.

In the second part of the model, a fully connected layer is applied, followed by batch normalization and dropout layers. The dropout layers are essential for preventing prevent overfitting. If additional features are used, such as mRNA half-life data or transcription factor target data, they are merged with the model before the fully connected layer.

The CNN in the model is configured with 64 channels and a kernel size of 3. The fully connected layer containes 64 parameters. Dropout rates are set to 0.6 for the first dropout layer, 0.4 for the second, and 0.2 for the third.

For comparison, we have also designed a model that uses word vectors from only one pre-trained model. The structure of this model is illustrated in Supplementary Figure S2b. The same hyperparameters are applied to this model, except for the dropout rates. In this case, the dropout rate is set to 0.6 for the first dropout layer and 0.2 for the second.

The experimental process and result of the ablation study is presented in Appendix B.

### 2.7. TExCNN Model with a 50,000-bp Input Length

When the input sequence is extended to 50,000 bp, the word embedding strategy is adjusted to accommodate the increased sequence length. The TExCNN model with the 50,000-bp input length uses four word embeddings as inputs:

1. Word vectors from the 10,000 bp upstream and 10,000 bp downstream sequences generated by DNABERT;
2. Word vectors from the 10,000 bp upstream and 10,000 bp downstream sequences generated by DNABERT-2;
3. Word vectors from the 25,000 bp upstream and 25,000 bp downstream sequences generated by DNABERT;
4. Word vectors from the 25,000 bp upstream and 25,000 bp downstream sequences generated by DNABERT-2.

Each input is passed through a CNN layer, followed by a batch normalization layer and a dropout layer, before being concatenated. The model structure is shown in Supplementary Figure S3.

For inputs 1 and 2, we use a kernel size of 3 and set the dropout rate to 0.6. For inputs 3 and 4, the kernel size is increased to 5, with a dropout rate of 0.9. All other hyperparameters remain the same as those used in the TExCNN model with a 10,500-bp input length.

## 3. Results

### 3.1. Experimental Settings

To evaluate the prediction ability of the proposed TExCNN framework, we conducted experiments under several different conditions. The primary goal was to assess the model’s performance in predicting gene expression levels from DNA sequences, using the coefficient of determination (R^2^) as the evaluation metric for the regression task.

The following evaluations were carried out. First, the performance of TExCNN was compared against the state-of-the-art models, DeepLncLoc and DNAPerceiver. The same dataset was used for training, validation, and testing to ensure a fair comparison. Second, TExCNN was tested using different pre-trained models, including DNABERT and DNABERT-2. Experiments were conducted with embeddings from each model individually and in combination to determine the optimal configuration. Third, to explore the effect of input sequence length on prediction performance, TExCNN was evaluated with two sequence lengths: 10,500 bp and 50,000 bp. The results were compared to assess how the extended sequence length impacts model performance. Finally, additional features, such as mRNA half-life data and transcription factor binding data, were incorporated into the model to examine their influence on predictive performance. Experiments were performed both with and without these additional features to evaluate their contribution.

For the 10,500 bp input sequence, the total number of training epochs was set to 50. For the 50,000 bp input sequence, the total number of training epochs was set to 100. The early stopping based on validation performance was applied, with a patience of 20 epochs. The batch size was set to 128. Adam was used as the optimizer during training, with a learning rate decay schedule. The initial learning rate was set to 0.002 and reduced to 0.0002 after ten epochs. The R^2^ metric was calculated on the test set to evaluate the performance of the models.

We also used the 10-fold validation method to assess whether TExCNN was overfitting on the test sets of the Xpresso dataset and DeepLncLoc dataset. The results of the 10-fold valiadation experiment are shown in Appendix C.

Finally, we trained and evaluated TExCNN on an independent dataset to evaluate the robustness of the model. The results of the test are shown in Appendix D.

### 3.2. Comparison Between TExCNN, DeepLncLoc, and DNAPerceiver

We first compared the performance of TExCNN with the model DeepLncLoc [15]. Both models were evaluated under three experimental conditions: (1) using only DNA sequences, (2) using DNA sequences and mRNA half-life features, and (3) using DNA sequences, mRNA half-life features, and transcription factor (TF) data. Each model was trained ten times for each condition, and the maximum, minimum, and average R^2^ values were reported from these runs.

To ensure a fair comparison, we replicated the experiments using the same datasets and conditions for TExCNN. Three configurations of TExCNN were evaluated: one using both DNABERT and DNABERT-2 embeddings, and two additional versions using either DNABERT or DNABERT-2 alone. The results for DeepLncLoc were taken from the original paper.

**Figure 2:**
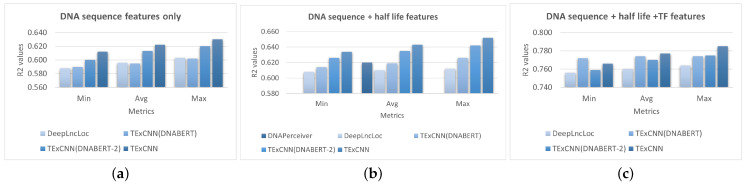
Performance comparison between DeepLncLoc and TExCNN. Different feature configurations are evaluated, including (**a**) DNA sequence features only, (**b**) DNA sequence and mRNA half-life features, and (**c**) DNA sequence, mRNA half-life, and TF features. The horizontal axis represents the minimum (Min), average (Avg), and maximum (Max) values of each model’s 10 independent runs. The vertical axis shows the R^2^ values.

As shown in Figure 2, TExCNN consistently outperformed DeepLncLoc across all conditions. The largest improvement was observed when using DNA sequence and mRNA half-life features, where TExCNN shows a 5.41% higher average R^2^ compared to DeepLncLoc (Figure 2b).

According to Ref. [10], the DNAPerceiver model achieved an R^2^ of 0.62 when using DNA sequence and mRNA half-life data as features, which is higher than the average R^2^ of DeepLncLoc and TExCNN(DNABERT). Howerver, the average R^2^ of TExCNN(DNABERT-2) and TExCNN still outperformed DNAPercevier, as shown in Figure 2b.

### 3.3. Evaluation of Different Feature Combinations

We evaluated the impact of different feature combinations on the performance of TExCNN, focusing on three scenarios: (a) using only DNA sequences, (b) using DNA sequences and mRNA half-life features, and (c) using DNA sequences, mRNA half-life features, and TF data.

TExCNN, using the full feature set (DNA sequence, mRNA half-life, and TF data), achieved the highest average R^2^ score of 0.777 (Figure 2c). This demonstrates that integrating multiple feature types provides complementary information and significantly improves the predictive performance of the model. Notably, TExCNN, even when using only DNA sequences, outperformed DeepLncLoc, which incorporated both DNA sequences and mRNA half-life features. Moreover, the performance of TExCNN was positively correlated with the number of feature types using in the model (Figure 2).

These results underscore the importance of incorporating additional biological data, such as mRNA half-life and transcription factor (TF) information, to improve the accuracy of gene expression prediction models.

### 3.4. Evaluation of Different Pre-Trained Model Combinations

To evaluate the contribution of different pre-trained models, we tested three configurations of TExCNN: (1) using word embeddings from both DNABERT and DNABERT-2, (2) using only DNABERT embeddings, and (3) using only DNABERT-2 embeddings. Across all feature combinations, TExCNN using both pre-trained models consistently consistently achieved the best performance, as shown in Figure 2. Even when only one pre-trained model was used, TExCNN outperformed DeepLncLoc in all the cases.

This demonstrates that the integration of pre-trained models enhances the model’s ability to capture hidden patterns in DNA sequences, leading to more accurate predictions of gene expression levels.

The combination of DNABERT and DNABERT-2 provides complementary information that strengthens the model’s predictive power, confirming that using multiple pre-trained models can further improve the performance of gene expression prediction tasks.

### 3.5. Evaluation of Extended Input Length

The proposed TExCNN model was also evaluated using the 50,000-bp input sequences to predict median gene expression values. The experiment was conducted under the same conditions as described in Section 3.1, with the model trained independently 10 times for each condition.

**Figure 3:**
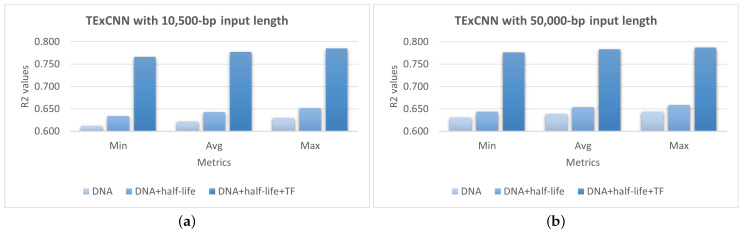
Evaluation of different feature combinations on the extended dataset. Different feature configurations have been evaluated, including (1) DNA sequence features only (DNA), (2) DNA sequence and mRNA half-life features (DNA + half-life), and (3) DNA sequence, mRNA half-life, and TF features (DNA + half-life + TF). The comparison is between the TExCNN models with (**a**) 10,500-bp and (**b**) 50,000-bp input lengths. The horizontal axis represents the minimum (Min), average (Avg), and maximum (Max) values of each model’s 10 independent runs. The experiment is conducted on the extended dataset with 50,000-bp sequence length. The vertical axis gives the R^2^ values.

As shown in Figure 3, TExCNN with the 50,000-bp input length consistently outperformed its version with 10,500-bp input length. When using only DNA sequences, the average R^2^ of TExCNN with the 50,000-bp input length was 0.017 higher than that of TExCNN with the 10,500-bp input length. When incorporating DNA sequences and mRNA half-life features, the improvement on the average R^2^ was 0.011. The R^2^ could still be improved by 0.006 when all the three feature types were used.

These results suggest that extending the input sequence length to 50,000 bp leads to further improvements in prediction performance across all feature combinations.

### 3.6. Application of TExCNN Models on Different Cells and Tissues

We further explored the ability of TExCNN to predict gene expression levels across different cells and tissues. In this experiment, we used 50,000-bp long DNA sequences, mRNA half-life features, and transcription factor data as input features, with expression levels serving as labels.

**Figure 4:**
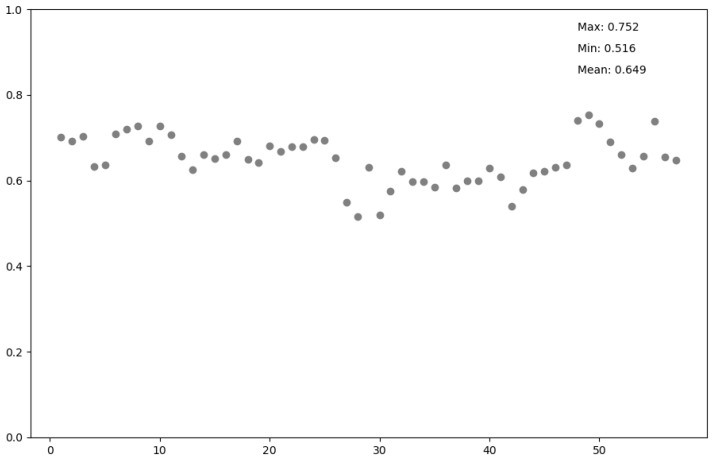
The R^2^ values of predicting gene expression levels on 57 cells and tissues.

Figure 4 shows that the TExCNN model achieved an average R^2^ of 0.649, with a maximum R^2^ of 0.752 and a minimum R^2^ of 0.516 across the 57 different cell and tissue types. Specifically, the model performed best on the prediction of A549, GM12878, HELA, and K562, where the R^2^ reached 0.739, 0.752, 0.732, and 0.739, respectively. It performed relatively worse on the prediction of Aorta, Adult Liver, Brain Hippocampus Middle, and Right Atrium, where the R^2^ reached 0.549, 0.516, 0.519, and 0.540, respectively. These results demonstrate that TExCNN performed robustly across various cell and tissue types, highlighting its versatility in predicting gene expression.

### 3.7. Which Part of the Sequence Affects Gene Regulation the Most

Finally, we investigated how different parts of the sequence contribute to prediction performance. Using the TExCNN structure and hyperparameters described in Section 2.6, we trained the model on four different datasets, which were:

1. The 3000-bp upstream and 3000-bp downstream sequences;
2. The 6000-bp upstream and 6000-bp downstream sequences;
3. The 10,000-bp upstream and 10,000-bp downstream sequences;
4. The 25,000-bp upstream and 25,000-bp downstream sequences.

**Figure 5:**
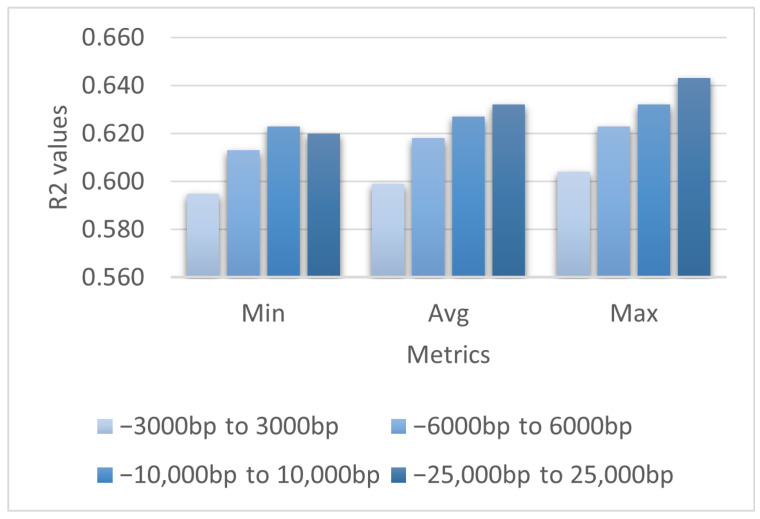
Evaluation of TExCNN trained on four different datasets. The horizontal axis represents the minimum (Min), average (Avg), and maximum (Max) values of each model’s 10 independent runs. The vertical axis gives the R^2^ values.

According to Figure 5, it is apparent that TExCNN performed better when the input sequence length was increased. TExCNN trained on 25,000-bp upstream and 25,000-bp downstream sequences reacheed an average R^2^ of 0.632, which was 0.033 higher than the model trained on 3000-bp upstream and 3000-bp downstream sequences.

This suggests that the sequence closer to the promoter, including coding DNA, plays a more significant role in gene regulation, while the more distal non-coding DNA has a relatively minor impact.

## 4. Discussion

This study proposed a novel framework, TExCNN, for predicting median gene expression levels using pre-trained models, DNABERT and DNABERT-2, to generate word embeddings from DNA sequences. Our results show that TExCNN has outperformed the state-of-the-art model, especially when incorporating longer sequences as features.

The study demonstrates that expanding sequence length to from 10,500 bp to 50,000 bp leads to better prediction performance. Furthermore, TExCNN with the 50,000 bp input length effectively has predicted expression values for different cell and tissue types, showcasing its versatility in both cell-type-independent and cell-type-dependent applications.

This study highlights some limitations of traditional fine-tuning approaches, such as handling long input sequences, high computational costs, and task specialization. In contrast, TExCNN addresses these challenges by bypassing the input length limit, reducing computational demands, and allowing reusable word embeddings for various tasks. This study also reveals the potential application of self-supervised learning (SSL) in the field of bioinfomatics [30].

Future research will focus on further improving the model’s performance and extending its applications to other species. Additionally, we plan to explore the role of long-range interactions in gene regulation and the use of other pre-trained models to capture more hidden information from genomic sequences.

## Figures and Tables

**Figure A1:**
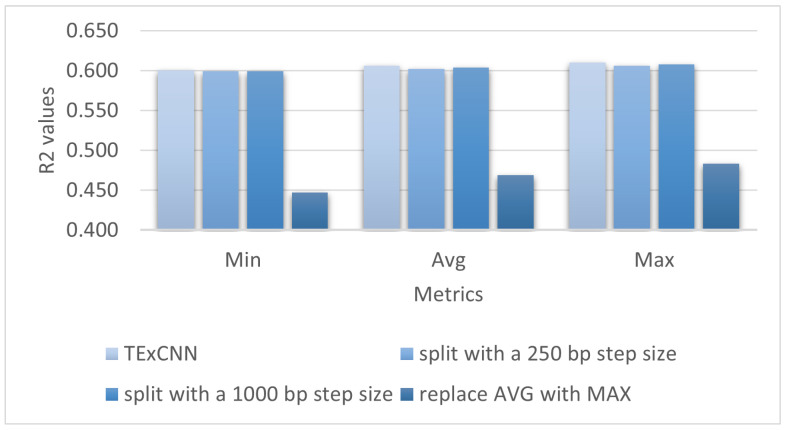
The comparison experiment between different pre-processing methods. The horizontal axis represents the minimum (Min), average (Avg), and maximum (Max) values of each model’s 10 independent runs. The vertical axis gives the R^2^ values.

**Figure A2:**
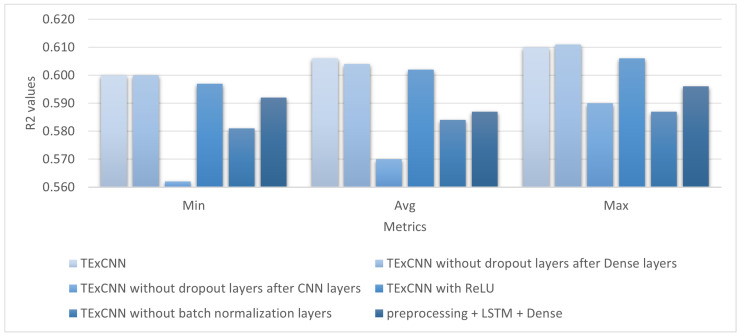
The ablation experiment of TExCNN. The horizontal axis represents the minimum (Min), average (Avg), and maximum (Max) values of each model’s 10 independent runs. The vertical axis gives the R^2^ values.

**Figure A3:**
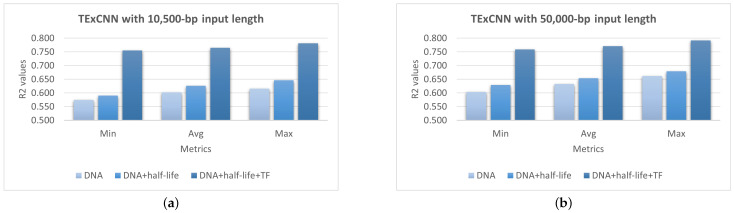
The results of the 10-fold validation test. Different feature configurations have been evaluated, including (1) DNA sequence features only (DNA), (2) DNA sequence and mRNA half-life features (DNA + half-life), and (3) DNA sequence, mRNA half-life, and TF features (DNA + half-life + TF). The comparison is between the TExCNN models with (**a**) 10,500-bp and (**b**) 50,000-bp input lengths. The horizontal axis represents the minimum (Min), average (Avg), and maximum (Max) values of each model’s 10 independent runs. The vertical axis gives the R^2^ values.

**Figure A4:**
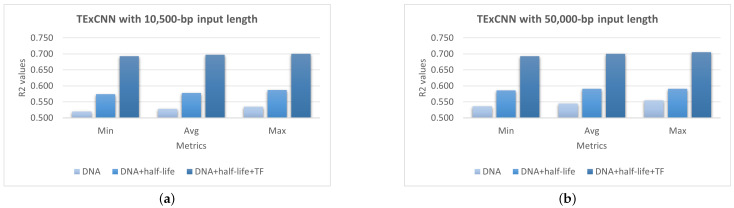
The results for the independent dataset. Different feature configurations have been evaluated, including (1) DNA sequence features only (DNA), (2) DNA sequence and mRNA half-life features (DNA + half-life), and (3) DNA sequence, mRNA half-life, and TF features (DNA + half-life + TF). The comparison is between the TExCNN models with (**a**) 10,500-bp and (**b**) 50,000-bp input lengths. The horizontal axis represents the minimum (Min), average (Avg), and maximum (Max) values of each model’s 10 independent runs. The vertical axis gives the R^2^ values.

## Data Availability

The source codes are available at https://healthinformaticslab.org/supp/, (accessed on 14 November 2024). The Xpresso dataset and the Deeplncloc dataset can be downloaded from https://github.com/vagarwal87/Xpresso, (accessed on 14 November 2024) and https://github.com/geneexpressionpolito/Predicting-gene-expression-levels-from-DNA-sequences-and-post-transcriptional-info-with-transformers, (accessed on 14 November 2024). The hg38 can be downloaded from http://hgdownload.soe.ucsc.edu/downloads.html, (accessed on 14 November 2024). The normalized expression values for protein-coding mRNAs across 57 tissues and cell lines (including 1 universal-human-reference expression) from RNA-seq data can be downloaded from https://egg2.wustl.edu/roadmap/data/byDataType/rna/expression/57epigenomes.RPKM.pc.gz, (accessed on 14 November 2024).

## Supplementary Materials

The following supporting information can be downloaded at: https://www.mdpi.com/article/10.3390/genes15121593/s1, Figure S1: Nucleotide sequence representations using the pre-trained models. The representation strategies for the (a) DNABERT and (b) DNABERT-2 are illustrated, respectively; Figure S2: Architectures of the TExCNN models with 10,500-bp input length. The TExCNN models are trained using (a) both pre-trained models and (b) only one pre-trained model; Figure S3: The structure of the TExCNN model with 50,000-bp input length; Figure S4: The structure of the LSTM model in Appendix D.

## Appendix A

We compared four different pre-processing methods, which were:

1. The methods in Section 2.5;
2. When leveraging DNABERT-2, splitting the sequence into fragments of 2000 bp with a 250 bp step size;
3. When leveraging DNABERT-2, splitting the sequence into fragments of 2000 bp with a 1000 bp step size;
4. Using the same splitting methods in Section 2.5 and replacing the average vectors of embeddings with the maximum vectors of embeddings.

We performed the comparison experiments on the Xpresso valiadation set. We used only the 10,500 bp length of DNA sequences as features. The other experimental settings were the same as the experiment settings in Section 3.1.

According to Figure A1, 500 bp is the best step size, and the averaging strategy is much better than the maximum strategy.

## Appendix B

In order to show the superiority of the model structure of TExCNN, we performed the ablation experiments on the Xpresso valiadation set. We used only the 10,500 bp length of DNA sequences as features. The other experimental settings were the same as the experiment settings in Section 3.1.

Addtionally, we compared the performance between TExCNN and the LSTM model. The LSTM model shared the same DNA sequence pre-processing as TExCNN. The units of LSTM was set to 128. The fully connected layer contained 64 parameters. Dropout rates were set to 0.6 for the first dropout layer and 0.2 for the second layer. The structure of the LSTM model can be seen in Supplementary Figure S4.

According to Figure A2, the dropout layers after the CNN layers and the batch normalization layers significantly contributed to the model’s predictive performance. Additionally, TExCNN outperformed the LSTM model in this comparison.

## Appendix C

We shuffled the original training, validation, and testing sets randomly and separated them into 10 pieces. We trained TExCNN on the new dataset ten times, using 80% of the dataset as the training set, 10% as the valiadation set, and 10% as the testing set each time. The results of the 10-fold validation test are shown in Figure A3.

## Appendix D

We downloaded the Human liver RNA-Seq gene expression from https://www.kaggle.com/datasets/lachmann12/human-liver-rnaseq-gene-expression-903-samples, (accessed on 2 December 2024) [31]. We extracted the genes that were in the Xpresso and DeepLncLoc dataset. We calculated the median of these expression values, converted them using the function y → $\log_{10}\left(y+0.1\right)$, and normalized them. The results of the 10-fold validation test are shown in Figure A4.
