# Supplementary material for: TExCNN: Leveraging Pre-Trained Models to Predict Gene Expression from Genomic Sequences
Source: Genes (Basel). 2024 Dec 12;15(12):1593. doi: 10.3390/genes15121593 (PMC11675716; doi:10.3390/genes15121593)

Supplementary Figure S1

Nucleotide sequence representations using the pre-trained models. The representation strategies for the (a) DNABERT and (b) DNABERT-2 are illustrated, respectively.

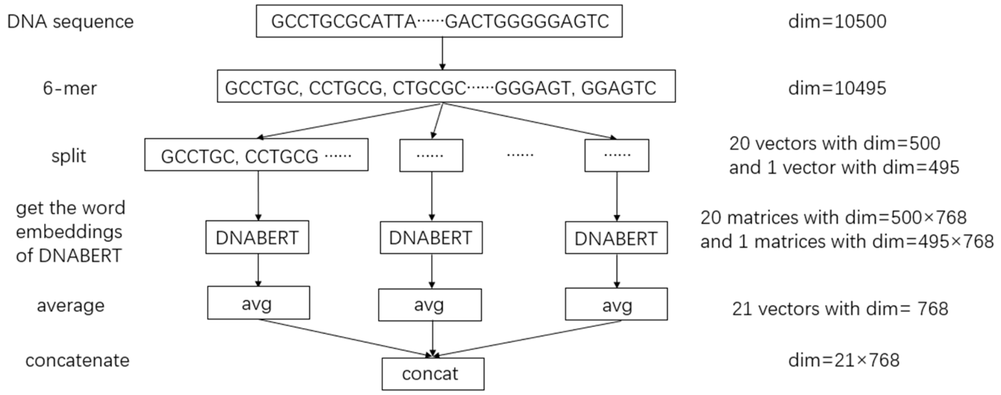

(a)

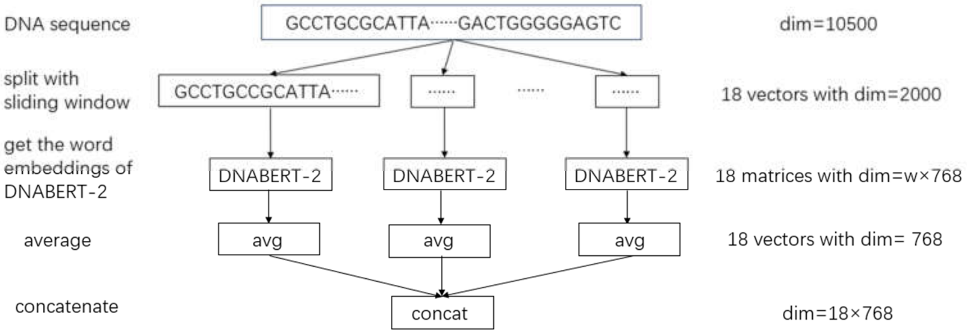

(b)

Supplementary Figure S2

Architectures of the TExCNN models with 10500-bp input length. The TExCNN models are trained using (a) both pre-trained models and (b) only one pre-trained model.

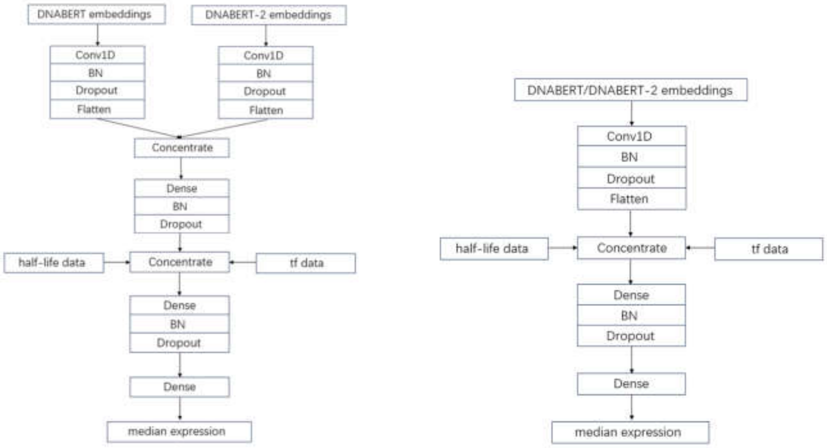

(a) (b)

Supplementary Figure S3

The structure of the TExCNN model with 50000-bp input length.

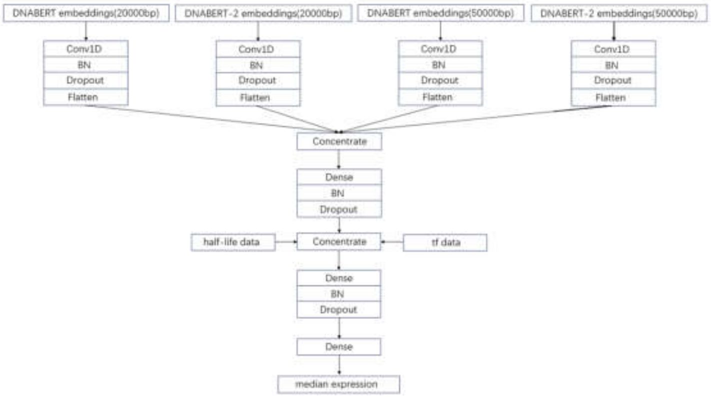

Supplementary Figure S4

The structure of the LSTM model in Appendix D.

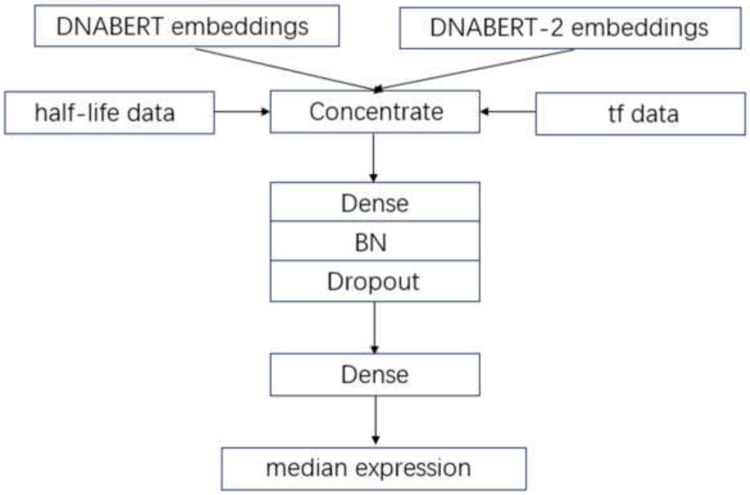

Supplement: Supplementary file 1 [file genes-15-01593-s001.zip › genes-3342798-supplementary.pdf]
